# Supplementary material for: The impacts of social restrictions during the COVID-19 pandemic on the physical activity levels of over 50-year olds: The CHARIOT COVID-19 Rapid Response (CCRR) cohort study
Source: PLoS One. 2023 Sep 26;18(9):e0290064. doi: 10.1371/journal.pone.0290064 (PMC10522032; doi:10.1371/journal.pone.0290064)

**S2**

S2.1 Causal diagrams for loneliness

Model 1


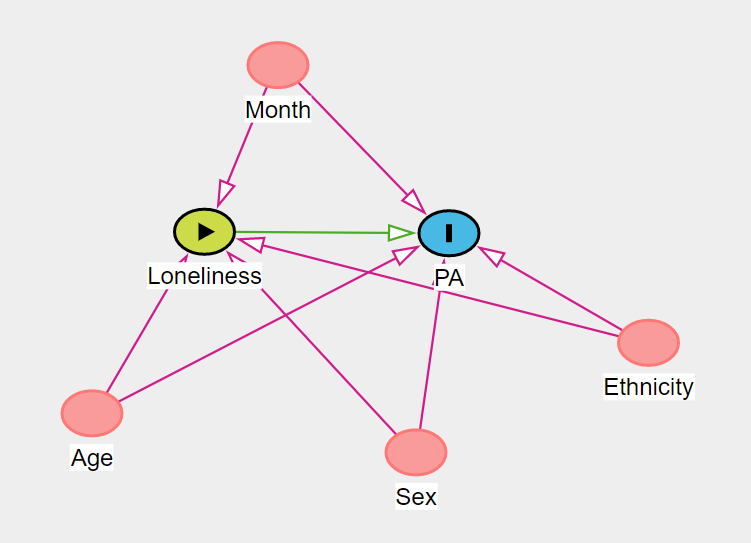


Model 2


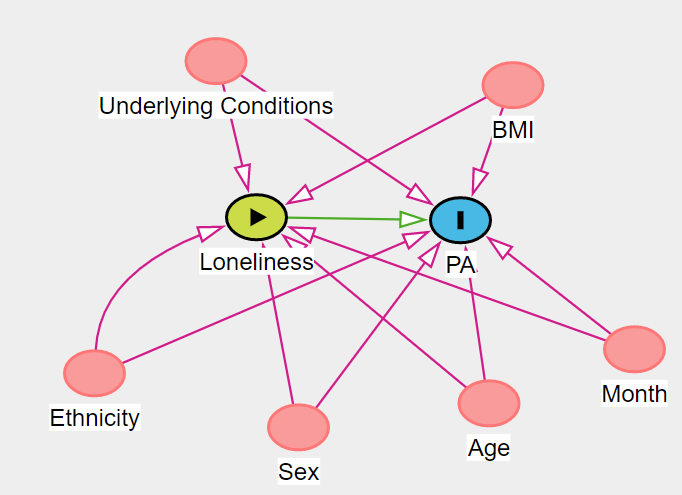


Model 3


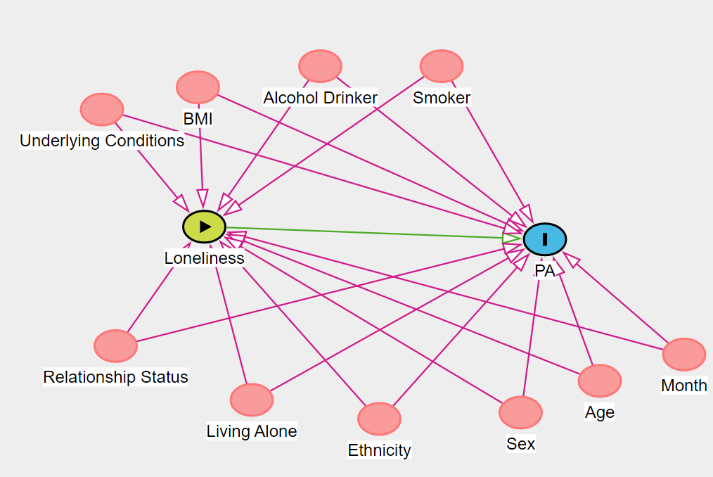


S2.2 Causal diagrams for shielding

Model 1


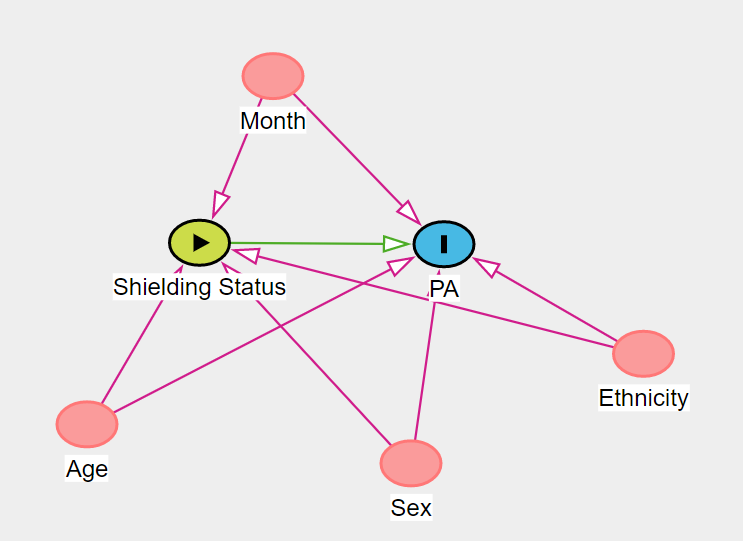


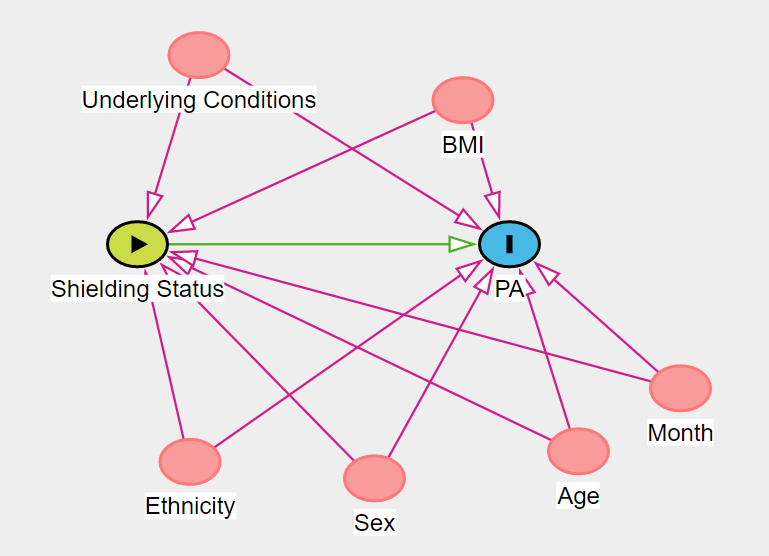
Model 2

Model 3


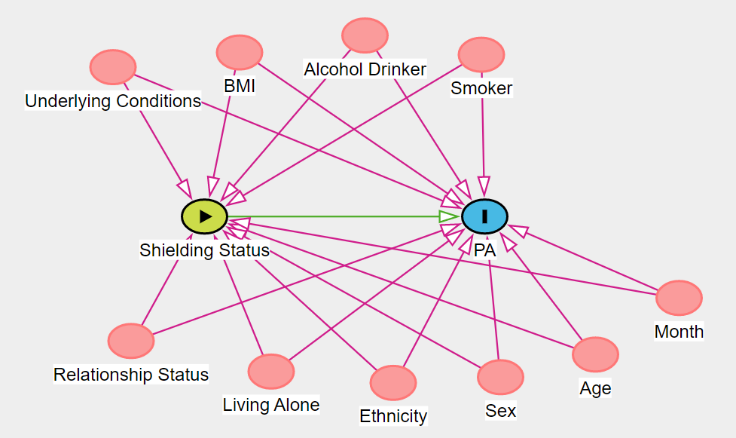

Supplement: S2 File — (DOCX) [file pone.0290064.s003.docx]
