# Supplementary material for: The impacts of social restrictions during the COVID-19 pandemic on the physical activity levels of over 50-year olds: The CHARIOT COVID-19 Rapid Response (CCRR) cohort study
Source: PLoS One. 2023 Sep 26;18(9):e0290064. doi: 10.1371/journal.pone.0290064 (PMC10522032; doi:10.1371/journal.pone.0290064)
Supplement: S3 File — (DOCX) [file pone.0290064.s004.docx]

**Supplementary File 3**

Model Equations

*Univariable:*

*yi,j= β0+ β 1Z+ µi*

*Model 1:*

*yi,j= β0+B1Zj+ β2age,j+ β3sex,j+ β4ethnicity,j+ β5Monthi.j + β6PA_prepandemic,j_ + µi*

*Model 2:*

*yi,j= β0+ β1Zj+ β2age,j+ β3sex,j+ β4ethnicity,j+ β5Monthi.j+ β6UnderlyingConditions,j+ β7BMI,j+* *β7PAprepandemic,j + µi*

*Model 3:*

*yi,j= β0+ β 1Zj+ β2age,j+ β3sex,j+ β4ethnicity,j+ β5Monthi.j+ β6UnderlyingConditions,j+ β7BMI,j+ β8AlcoholDrinker,j+ β9Smoker,j+ β10LivingAlone+ β11RelationshipStatus+ β12PA_prepandemic,j_ + µi*

Where i is survey wave in participant j, Z is the exposure, and y is PA
