# Supplementary material for: The impacts of social restrictions during the COVID-19 pandemic on the physical activity levels of over 50-year olds: The CHARIOT COVID-19 Rapid Response (CCRR) cohort study
Source: PLoS One. 2023 Sep 26;18(9):e0290064. doi: 10.1371/journal.pone.0290064 (PMC10522032; doi:10.1371/journal.pone.0290064)
Supplement: S4 File — (DOCX) [file pone.0290064.s005.docx]

**Supplementary File 4**

Coefficients of study variables and numbers of model observations and groups

| **Shielding Univariable** | | | **Loneliness Univariable** | | |
| --- | --- | --- | --- | --- | --- |
| Number of observations | 29416 | | Number of observations | 28694 | |
| Number of groups | 7326 | | Number of groups | 7011 | |
|  |  |  |  |  |  |
| **Predictor** | **Coeff** | **p** | **Predictor** | **Coeff** | **p** |
| Shielding | -352.35 | <0.001 | Often Lonely | -522.35 | <0.001 |
| Not Shielding (ref) | 0 |  | Sometimes Lonely | -95.12 | 0.1 |
|  |  |  | Rarely Lonely | -1.64 | 0.97 |
|  |  |  | Never Lonely (ref) | 0 |  |

| **Shielding model 1** | | | **Loneliness model 1** | | |
| --- | --- | --- | --- | --- | --- |
| Number of observations | 29214 | | Number of observations | 28498 | |
| Number of groups | 7268 | | Number of groups | 6956 | |
|  |  |  |  |  |  |
| **Predictor** | **Coeff** | **p** | **Predictor** | **Coeff** | **p** |
| Age | -13.45 | <0.001 | Age | -17.58 | <0.001 |
| Sex Male | 82.56 | 0.007 | Sex Male | 66.58 | 0.038 |
| Sex prefer not to say | 290.57 | 0.680 | Sex prefer not to say | 330.65 | 0.639 |
| Ethnicity Asian | -277.95 | 0.003 | Ethnicity Asian | -279.82 | 0.003 |
| Ethnicity Black African, Caribbean or Black British | -328.33 | 0.076 | Ethnicity Black African, Caribbean or Black British | -416.90 | 0.029 |
| Ethnicity Mixed or Multiple ethnic groups | -182.73 | 0.141 | Ethnicity Mixed or Multiple ethnic groups | -180.35 | 0.152 |
| Ethnicity Other ethnic group | -310.59 | 0.037 | Ethnicity Other ethnic group | -292.20 | 0.055 |
| Ethnicity Prefer not to say | -367.55 | 0.275 | Ethnicity Prefer not to say | -361.48 | 0.315 |
| Pre Pandemic MET min | 0.41 | <0.001 | Pre Pandemic MET min | 0.41 | <0.001 |
| Month Jun | -168.32 | 0.000 | Month Jun | -180.77 | <0.001 |
| Month Jul | -106.23 | 0.001 | Month Jul | -119.05 | <0.001 |
| Month Aug | -740.67 | 0.000 | Month Aug | -732.98 | <0.001 |
| Month Sep | -226.50 | 0.000 | Month Sep | -240.83 | <0.001 |
| Month Oct | -911.17 | 0.150 | Month Oct | -906.33 | 0.151 |
| Month Nov | -1275.55 | 0.481 | Month Nov | -1179.59 | 0.515 |
| Month Dec | -330.09 | <0.001 | Month Dec | -347.17 | <0.001 |
| Month Jan | -666.70 | 0.001 | Month Jan | -617.91 | 0.004 |
| Month Feb | -526.86 | 0.545 | Month Feb | -471.89 | 0.588 |
| Month Mar | -307.9 | <0.001 | Month Mar | -327.61 | <0.001 |
| Month Apr | -133.88 | 0.350 | Month Apr | -45.00 | 0.763 |
| Shielding | -228.20 | <0.001 | Often Lonely | -546.70 | <0.001 |
| Not Shielding (ref) | 0 |  | Sometimes Lonely | -111.80 | 0.04 |
|  |  |  | Rarely Lonely | -15.50 | 0.7 |
|  |  |  | Never Lonely (ref) | 0 |  |

| **Shielding model 2** | | | **Loneliness model 2** | | |
| --- | --- | --- | --- | --- | --- |
| Number of observations | 9443 | | Number of observations | 9257 | |
| Number of groups | 2551 | | Number of groups | 2456 | |
|  |  |  |  |  |  |
| **Predictor** | **Coeff** | **p** | **Predictor** | **Coeff** | **p** |
| Age | -9.55 | 0.007 | Age | -10.69 | 0.003 |
| Sex Male | 110.04 | 0.040 | Sex Male | 90.56 | 0.104 |
| Sex prefer not to say | 1377.86 | 0.297 | Sex prefer not to say | 1247.53 | 0.345 |
| Ethnicity Asian | -119.34 | 0.399 | Ethnicity Asian | -87.61 | 0.546 |
| Ethnicity Black African, Caribbean or Black British | -308.64 | 0.246 | Ethnicity Black African, Caribbean or Black British | -314.49 | 0.245 |
| Ethnicity Mixed or Multiple ethnic groups | 3.05 | 0.990 | Ethnicity Mixed or Multiple ethnic groups | 12.8 | 0.959 |
| Ethnicity Other ethnic group | -357.87 | 0.252 | Ethnicity Other ethnic group | -356.45 | 0.254 |
| Ethnicity Prefer not to say | -1217.6 | 0.244 | Ethnicity Prefer not to say | -1245.85 | 0.490 |
| Pre Pandemic MET min | 0.38 | <0.001 | Pre Pandemic MET min | 0.38 | <0.001 |
| Month Jun | -170.68 | 0.002 | Month Jun | -181.8 | 0.001 |
| Month Jul | -202.69 | <0.001 | Month Jul | -215.22 | <0.001 |
| Month Aug | -959 | <0.001 | Month Aug | -957.66 | <0.001 |
| Month Sep | -325.3 | <0.001 | Month Sep | -329.88 | <0.001 |
| Month Oct | -1187.55 | 0.083 | Month Oct | -1186.92 | 0.083 |
| Month Dec | -433.25 | <0.001 | Month Dec | -443.94 | <0.001 |
| Month Jan | -612.12 | 0.010 | Month Jan | -575.73 | 0.021 |
| Month Feb | -838.24 | 0.415 | Month Feb | -764.32 | 0.458 |
| Month Mar | -439.57 | <0.001 | Month Mar | -451.1 | <0.001 |
| BMI | -16.36 | 0.001 | BMI | -17.2 | 0.001 |
| Underlying conditions: prefer not to say | -702.3 | 0.421 | Underlying conditions: prefer not to say | -692.64 | 0.428 |
| Underlying conditions: yes | -194.41 | <0.001 | Underlying conditions: yes | -199.19 | <0.001 |
| Shielding | -113.2 | 0.11 | Often Lonely | -83.81 | 0.71 |
| Not Shielding (ref) | 0 |  | Sometimes Lonely | -46.89 | 0.6 |
|  |  |  | Rarely Lonely | -51.35 | 0.44 |
|  |  |  | Never Lonely (ref) | 0 |  |

| **Shielding model 3** | | | **Loneliness model** | | |
| --- | --- | --- | --- | --- | --- |
| Number of observations | 8798 | | Number of observations | 8625 | |
| Number of groups | 2404 | | Number of groups | 2312 | |
|  |  |  |  |  |  |
| **Predictor** | **Coeff** | **p** | **Predictor** | **Coeff** | **p** |
| Age | -13.46 | 0.001 | Age | -13.91 | 0.001 |
| Sex Male | 65.5 | 0.302 | Sex Male | 59.3 | 0.363 |
| Sex prefer not to say | 1331.88 | 0.361 | Sex prefer not to say | 1213.72 | 0.406 |
| Ethnicity Asian | -220.12 | 0.172 | Ethnicity Asian | -175.9 | 0.290 |
| Ethnicity Black African, Caribbean or Black British | -389.61 | 0.193 | Ethnicity Black African, Caribbean or Black British | -402.42 | 0.186 |
| Ethnicity Mixed or Multiple ethnic groups | 34.81 | 0.900 | Ethnicity Mixed or Multiple ethnic groups | 21.41 | 0.939 |
| Ethnicity Other ethnic group | -406.75 | 0.287 | Ethnicity Other ethnic group | -413.43 | 0.280 |
| Ethnicity Prefer not to say | -1152.32 | 0.294 | Ethnicity Prefer not to say | -1691.08 | 0.373 |
| Pre Pandemic MET min | 0.29 | <0.001 | Pre Pandemic MET min | 0.29 | <0.001 |
| Month Jun | 269.28 | <0.001 | Month Jun | 263.59 | <0.001 |
| Month Jul | 402.01 | <0.001 | Month Jul | 388.4 | <0.001 |
| Month Aug | -271.47 | 0.074 | Month Aug | -261.85 | 0.091 |
| Month Sep | 343.36 | <0.011 | Month Sep | 340.99 | <0.001 |
| Month Oct | -396.28 | 0.566 | Month Oct | -400.87 | 0.561 |
| Month Dec | 255.74 | <0.001 | Month Dec | 247.26 | 0.001 |
| Month Jan | -520.27 | 0.041 | Month Jan | -481.1 | 0.074 |
| Month Feb | -1365.15 | 0.309 | Month Feb | -1382.39 | 0.304 |
| Month Mar | 245.61 | 0.001 | Month Mar | 236.89 | 0.001 |
| BMI | -27.19 | <0.001 | BMI | -28.28 | <0.001 |
| Underlying conditions: prefer not to say | -1140.04 | 0.230 | Underlying conditions: prefer not to say | -1187 | 0.212 |
| Underlying conditions: yes | -299.87 | <0.001 | Underlying conditions: yes | -305.38 | <0.001 |
| Smoker: yes | 55.54 | 0.742 | Smoker: yes | 45.07 | 0.790 |
| Alcohol Drinker: Yes | 151.79 | 0.055 | Alcohol Drinker: Yes | 160.18 | 0.046 |
| living alone: yes | 164.85 | 0.130 | living alone: yes | 163.69 | 0.139 |
| Relationship Status: single | -257.8 | 0.014 | Relationship Status: single | -226.38 | 0.035 |
| Shielding | -98.41 | 0.19 | Often Lonely | -10.64 | 0.96 |
| Not Shielding (ref) | 0 |  | Sometimes Lonely | 3.77 | 0.97 |
|  |  |  | Rarely Lonely | -30.10 | 0.67 |
|  |  |  | Never Lonely (ref) | 0 |  |
